# Psychometric Properties of the EQ-5D for the Assessment of Health-Related Quality of Life in the Population of Middle-Old and Oldest-Old Persons: Study Protocol for a Systematic Review

**DOI:** 10.3389/fpubh.2020.578073

**Published:** 2020-10-30

**Authors:** Sophie Gottschalk, Hans-Helmut König, Mona Nejad, Judith Dams

**Affiliations:** Department of Health Economics and Health Services Research, University Medical Center Hamburg-Eppendorf, Hamburg, Germany

**Keywords:** systematic review, psychometric properties, oldest-old population, older population, EQ-5D

## Abstract

**Introduction:** Health care interventions for middle-old and oldest-old individuals (75 years or older) are often economically evaluated using the EuroQol questionnaire (EQ-5D) to measure health-related quality of life. However, the psychometric performance of the EQ-5D in this population has been questioned, as it probably does not adequately capture relevant aspects of quality of life in the older population. Because the results of economic evaluations using the EQ-5D often guide decision-makers, it is important to know whether the EQ-5D has satisfactory psychometric properties in the middle-old and oldest-old population. Therefore, studies assessing the psychometric properties of the EQ-5D in this population should be synthesized by a systematic review.

**Methods and Analysis:** A systematic review of studies providing empirical evidence of reliability, validity, and/or responsiveness of the EQ-5D in a sample with a mean age ≥75 years will be conducted. The databases PubMed, Web of Science, and EconLit will be searched. In addition, reference lists of included studies will be hand-searched. Two independent reviewers will select studies and assess their risk of bias with the COnsensus-based Standards for the selection of health Measurement Instruments (COSMIN) Risk of Bias checklist. Relevant data will be extracted by one reviewer and cross-checked by a second reviewer. Potential disagreements in any phase will be resolved through discussion with a third person. The guidelines for systematic reviews of measurement properties proposed by the COSMIN group, including criteria of good measurement properties, will guide the synthesis and interpretation of the results.

**Discussion:** The review's results could facilitate the making of recommendations for the use of the EQ-5D in a population of middle-old and oldest-old people and thereby being of interest for decision-makers or for researchers designing new intervention studies for older people. Heterogeneity of individual studies regarding the population under study could limit the possibility of making a synthesized statement on the appropriateness of the EQ-5D for the middle-old to oldest-old population.

## Introduction

In the context of demographic change, the older population, especially the population of middle-old and oldest-old (75 years or older), is increasing (1). As this population has typically a high number of (co)morbidities, a range of health care interventions aiming to improve the health and quality of life (QoL) of older persons has been developed. However, given the scarcity of resources, economic evaluations of new interventions are crucial for decision-making regarding their implementation as they provide information on the efficient allocation of resources. In economic evaluations, effectiveness of interventions is often measured by health-related QoL (HrQoL). In order to make effects comparable across interventions, generic instruments of HrQoL are used.

The most frequently used generic instrument of HrQoL in economic evaluations is the EuroQol 5 dimensions questionnaire (EQ-5D) (2). The advantage of the EQ-5D is its brevity and easy administration by consisting of only five questions covering the dimensions mobility, self-care, usual activities, pain/discomfort, and anxiety/depression. Depending on the version of the EQ-5D, each dimension has three (EQ-5D-3L) or five (EQ-5D-5L) severity levels. Despite its brevity, it is important that the EQ-5D is psychometrically sound in the population it is used, meaning that it measures what it intended to measure (validity) in an accurate and reproducible way (reliability) and is able to detect small but important changes over time (responsiveness). In the absence of sufficient psychometric properties, the results of economic evaluations of interventions fail in measuring the true effect of interventions on HrQoL and thus are not suitable as basis for decision-making regarding their implementation.

The approach to primarily focus on HrQoL in the form of health utility gains in economic evaluations has been criticized as it excludes aspects of QoL beyond health. As people's needs and desires change with age, significant intervention effects beyond the health status may not be sufficiently captured for the middle-old and oldest-old. Therefore, other instruments than the EQ-5D with a different theoretical approach have been developed. One example are the ICECAP instruments (3), which were developed based on the capability theory (4). Contrary to HrQoL, capability focuses on the *ability* of a person to function and not on *functioning*. With the ICECAP-O, an instrument has been developed especially for the assessment of QoL in older people (5). The development was based on in-depth interviews with the aim of identifying *attributes* of QoL instead of only *influences* on QoL. In this context, health was seen as an influence on attributes rather than as an attribute on its own (6). Especially at the end of life, it was shown that it is not appropriate to apply an exclusively health-focused perspective in economic evaluations, because aspects that go beyond health (e.g., choice/having a say, being with people who care, dignity, and preparation) become more important (7).

Nevertheless, the EQ-5D is still the most widely used instrument for economic evaluations, and as it aims to measure HrQoL, its validity cannot be judged by the fact that it does not capture factors beyond health. Previous reviews have been conducted regarding the psychometric performance of the EQ-5D focusing on different population groups. The EQ-5D was found appropriate for depression and personality disorders (8, 9), urinary incontinence (10), some skin diseases (11), and in people 60 years or older (12). However, the EQ-5D lacked psychometric performance in populations with anxiety, schizophrenia, and bipolar disorders, as well as in those with multiple sclerosis (8, 9, 13). Moreover, Tordrup et al. (14) evaluated the responsiveness of the EQ-5D in various disorders and concluded that the instrument is not sensitive to change in a range of disorders. Regarding the use of the EQ-5D in dementia, the validity was found problematic as there are significant disagreements between patient and proxy ratings and as the EQ-5D does not capture aspects that are particularly important for people with dementia (15, 16). Similarly, other authors conclude that the EQ-5D may not be appropriate in other conditions prevalent in the older population, such as hearing impairments, visual disorders, and some cancers (17, 18).

These findings, together with the literature on the capability approach that shifts the focus away from a mere health-utility perspective, raise questions regarding the appropriateness of the EQ-5D in a population of middle-old and oldest-old people. Because the results of economic evaluations using the EQ-5D as effect measure are often considered when deciding on the implementation of interventions targeting the middle-old and oldest-old population, it is important to know whether the EQ-5D has satisfactory psychometric properties in this population.

### Objective

This article provides the protocol for a systematic review that aims to synthesize and critically appraise studies assessing the psychometric properties of the EQ-5D in a population of middle-old and oldest-old people. Of interest are all studies reporting on reliability, validity, or responsiveness of the EQ-5D in a study population with a mean age of at least 75 years.

## Methods and Analysis

This protocol was based on the Preferred Reporting Items for Systematic Reviews and Meta-Analysis Protocols (PRISMA-P) (19) and will be registered in PROSPERO (registration not yet completed/currently being assessed).

### Eligibility Criteria

Cross-sectional or observational studies providing empirical evidence of reliability, validity, and/or responsiveness of the EQ-5D in a sample with a mean age of at least 75 years will be included. Included studies shall be published in peer-reviewed journals in German or English languages. Systematic reviews, studies applying a qualitative design, or studies being published in forms other than original articles (e.g., conference abstracts or comments) will be excluded. Furthermore, studies relying on proxy assessments only or those with the single objective of investigating agreement between different modes of administration of the EQ-5D will be excluded (e.g., studies only examining inter-rater agreement between the older person and a proxy). The question of inter-rater agreement between the patient and a proxy often concerns people with dementia and has been the subject of previous reviews (15, 16). There will be no restrictions relating to interventions, comorbidities/health conditions, publication date, or the version of the EQ-5D (three-level or five-level version).

### Information Sources and Search Strategy

PubMed, Web of Science, and EconLit will be searched electronically in August 2020 using predefined search terms, including EQ-5D, EuroQoL, aged, elder^*^, old^*^, geriatric^*^, *ag(e)ing*, and an adapted search filter for finding studies on measurement properties (20). This filter was developed to account for the large variation in terminology for measurement properties and unreliable indexing of studies under specific index terms, making it difficult to find all relevant studies under a small set of search terms (20). Because, for example, studies focusing on proxy assessments or interrater agreement only will be excluded from the planned review, search terms covering nonrelevant measurement properties (e.g., inter-rater reliability) were removed from the search filter. Where possible, search terms will be used as keywords in the title/abstract or Medical Subject Headings (MeSH terms). An example for the search strategy in PubMed is displayed in Table 1. Depending on the specific requirements of each database, the search terms will be modified. Additionally, the reference lists of included studies and previous reviews on HrQoL for middle-old and oldest-old people will be hand-searched.

**Table 1:** Search strategy in PubMed using an adapted version of the patient-reported outcome measurement filter available on the COSMIN website (20).

1#	(instrumentation[MeSH Subheading] OR “reproducibility of results”[MeSH Terms] OR reproducib*[Title/Abstract] OR “psychometrics”[MeSH] OR psychometr*[Title/Abstract] OR “discriminant analysis”[MeSH] OR reliab*[Title/Abstract] OR valid*[Title/Abstract] OR “internal consistency”[Title/Abstract] OR (cronbach*[Title/Abstract] AND (alpha[Title/Abstract] OR alphas[Title/Abstract])) OR “item correlation”[Title/Abstract] OR “item correlations”[Title/Abstract] OR agreement[Text Word] OR test–retest [Title/Abstract] OR (test[Title/Abstract] AND retest[Title/Abstract]) OR (reliab*[Title/Abstract] AND (test[Title/Abstract] OR retest[Title/Abstract])) OR intra-rater[Title/Abstract] OR intratester[Title/Abstract] OR intra-tester[Title/Abstract] OR OR intraobserver[Title/Abstract] OR intra-observer[Title/Abstract] OR intraindividual[Title/Abstract] OR intra-individual[Title/Abstract] OR intraparticipant[Title/Abstract] OR intra-participant[Title/Abstract] OR kappa[Title/Abstract] OR kappa's[Title/Abstract] OR kappa's[Title/Abstract] OR “coefficient of variation”[Title/Abstract] OR repeatable*[Text Word] OR ((replica*[Text Word] OR repeated[Text Word]) AND (measure[Text Word] OR measures[Text Word] OR findings[Text Word] OR result[Text Word] OR results[Text Word] OR test[Text Word] OR tests[Text Word])) OR concordance[Title/Abstract] OR (infraclass[Title/Abstract] AND correlation*[Title/Abstract]) OR discriminative[Title/Abstract] OR “known group” [Title/Abstract] OR “factor analysis”[Title/Abstract] OR “factor analyses”[Title/Abstract] OR “factor structure”[Title/Abstract] OR “factor structures”[Title/Abstract] OR dimensionality[Title/Abstract] OR subscale*[Title/Abstract] OR “item discriminant”[Title/Abstract]OR “interstate correlation”[Title/Abstract] OR “interstate correlations”[Title/Abstract] OR “individual variability”[Title/Abstract] OR “standard error of measurement”[Title/Abstract] OR sensitive*[Title/Abstract] OR responsive*[Title/Abstract] OR “minimal detectable concentration”[Title/Abstract] OR (small*[Title/Abstract] AND (real[Title/Abstract] OR detectable[Title/Abstract]) AND (change[Title/Abstract] OR difference[Title/Abstract])) OR “meaningful change”[Title/Abstract] OR “minimal important change”[Title/Abstract] OR “minimal important difference”[Title/Abstract] OR “minimally important change”[Title/Abstract] OR “minimally important difference”[Title/Abstract] OR “minimal detectable change”[Title/Abstract] OR “minimal detectable difference”[Title/Abstract] OR “minimally detectable change”[Title/Abstract] OR “minimally detectable difference”[Title/Abstract] OR “minimal real change”[Title/Abstract] OR “minimal real difference”[Title/Abstract] OR “minimally real change”[Title/Abstract] OR “minimally real difference”[Title/Abstract] OR “Item response model”[Title/Abstract] OR IRT[Title/Abstract] OR Rash[Title/Abstract] OR “Differential item functioning”[Title/Abstract] OR DIF[Title/Abstract])
#2	(EQ-5D) OR (EQ5D) OR (EuroQoL)
#3	(aged, 80 and over[MeSH Terms]) OR (aged[MeSH Terms]) OR (elderly[MeSH Terms]) OR (aged[Title/Abstract]) OR (elderly*[Title/Abstract]) OR (older*[Title/Abstract]) OR (geriatric*[Title/Abstract])
#4	#1 AND #2 AND #3
#5	(“addresses”[Publication Type] OR “biography”[Publication Type] OR “case reports”[Publication Type] OR “comment”[Publication Type] OR “directory”[Publication Type] OR “editorial”[Publication Type] OR “festschrift”[Publication Type] OR “interview”[Publication Type] OR “lectures”[Publication Type] OR “legal cases”[Publication Type] OR “legislation”[Publication Type] OR “letter”[Publication Type] OR “news”[Publication Type] OR “newspaper article”[Publication Type] OR “patient education handout”[Publication Type] OR “popular works”[Publication Type] OR “congresses” [Publication Type] OR “consensus development conference”[Publication Type] OR “consensus development conference, nigh”[Publication Type] OR “practice guideline”[Publication Type]) NOT (“animals”[MeSH Terms] NOT “humans”[MeSH Terms])
#6	#4 NOT #5

### Study Records (Data Management, Selection, and Collection)

Search results from all databases will be combined in a shared data repository and managed with the software Endnote X8. After removing duplicates, two independent reviewers (SG and MN) will screen the titles and abstracts for eligibility. Next, full texts of the selected abstracts will be assessed for eligibility by SG and MN independently. In case of disagreement or uncertainty, a third person (JD) will be consulted.

Using a standardized data extraction sheet, relevant data from the eligible studies will be extracted by one reviewer (SG) and cross-checked by the second reviewer (MN). Data extracted from the individual studies will include setting/country, population characteristics [sample size, distribution of age and sex, information on comorbidities (e.g., people with dementia)], instrument administration, type and method of validity, reliability and responsiveness assessment, and results of psychometric tests. The study selection process will be visualized in the form of a PRISMA flowchart.

### Data Items

The review's main outcomes of interest will be the results regarding validity, reliability, and responsiveness of the EQ-5D reported by the individual studies. Regarding the outcomes, we adhere to the taxonomy and definitions from the COnsensus-based Standards for the selection of health Measurement Instruments project (COSMIN) (21). According to the COSMIN group, reliability refers to the degree to which the measurement is free from measurement error and can be differentiated between internal consistency, reliability, and measurement error. Validity is referred to as the degree to which an instrument measures the construct(s) it purports to measure and consists of the subtypes content validity, construct validity, and criterion validity. Responsiveness is defined as the ability of an instrument to detect change over time in the construct to be measured.

### Assessment of Study Quality/Risk of Bias

Methodological quality of included studies will be assessed by the COSMIN Risk of Bias checklist, which has specifically been developed for use in systematic reviews of patient-reported outcome measures (22). It consists of 10 boxes, each referring to a particular measurement property and containing a different number of sub-questions. Each item is rated on a four-point scale, reaching from “very good” to “inadequate” (a “not applicable” option is also included). For each measurement property, an overall score will be determined by taking the lowest rating of any standard in the box (“worst score counts” principle). The checklist will be filled out by SG and MN independently. Any disagreements will again be resolved through discussion with a third person (JD).

### Data Synthesis

Based on criteria of good measurement properties (23, 24), the results of the individual studies will be rated as either “sufficient” (+), “insufficient” (–), or “indeterminate” (?). The individual studies' results will then be summarized, and an overall rating of the measurement property will be assigned. The results will be presented in a thematic order by structuring the results section in the following sub-sections: validity, reliability, and responsiveness. Each sub-section will be further divided into sections on different types of reliability or validity (e.g., content validity, construct validity, criterion validity). If necessary, e.g., in case of inconsistencies between different study populations, the results will be presented separately for different population groups (e.g., validity in people with dementia) or versions of the EQ-5D (EQ-5D-3L and EQ-5D-5L). The guidelines for systematic reviews of measurement properties proposed by the COSMIN group (25) will guide the synthesis and interpretation of the results.

## Discussion

This review aims to provide a summary statement on the appropriateness of using the EQ-5D in the middle-old and oldest-old population by summarizing the evidence regarding the validity, reliability, and responsiveness. Although previous reviews had a similar aim, they either focused on the psychometric properties of the EQ-5D in people with dementia only (15, 16) or are outdated and were not specifically focusing on the population of middle-old and oldest-old (12). The planned review could identify gaps in research that should be addressed by future studies. Furthermore, recommendations for the use of the EQ-5D in a population of middle-old and oldest-old people could be made based on the results of the review. For example, the review may conclude that, in addition to the EQ-5D, age- or disease-specific instruments should be used to better capture the specific needs and experiences of older people or specific subgroups of older people. Thereby, the results may be of interest not only for decision-makers, but also for researchers planning or designing new intervention studies for older people.

Potential limitations may arise because of the heterogeneity of the individual studies regarding the population under study (e.g., people with dementia, people with femoral fractures), which may limit the possibility of making a synthesized statement on the appropriateness of the EQ-5D for the middle-old to oldest-old population. The expected heterogeneity in study design, measurements used, and populations further precludes the possibility of performing a meta-analysis. Moreover, it may not be possible to make a statement exclusively for the population 75 years or older as there seems to be a lack of studies focusing exclusively on this population. Therefore, the inclusion criteria have been adapted to a mean age of the sample of at least 75 years, which may lead to the inclusion of a number of persons younger than 75 years.

## Author Contributions

The study concept was developed by SG, JD, and H-HK. The manuscript of the protocol was drafted by SG and critically revised by JD, H-HK, and MN. The search strategy was developed by SG and JD. Study selection, data extraction, and quality assessment will be performed by SG and MN, with JD as a third party in case of disagreements. All authors contributed to the article and approved the submitted version.

## Conflict of Interest

The authors declare that the research was conducted in the absence of any commercial or financial relationships that could be construed as a potential conflict of interest.

## Abbreviations

HrQoL: health-related quality of life
COSMIN: COnsensus-based Standards for the selection of health Measurement Instruments project.

## References

[1] United Nations, Department of Economic and Social Affairs, Population Division World Population Prospects 2019, Volume II: Demographic Profiles. New York, NY (2019).

[2] EuroQol G. EuroQol–a new facility for the measurement of health-related quality of life. Health Policy. (1990) 16:199–208. 10.1016/0168-8510(90)90421-9 10109801

[3] ICECAP Capability Measures. Available online at: https://www.birmingham.ac.uk/research/activity/mds/projects/HaPS/HE/ICECAP/index.aspx (accessed June 06, 2020).

[4] Sen A Choice, Welfare and Measurement. Cambridge, MA: Harvard University Press (1982).

[5] Coast J Flynn T Grewal I Lewis J Natarajan L Sproston K Developing an index of capability for older people: a new form of measure for public health interventions? In: Dawson S Morris ZS editors. Future Public Health. London: Palgrave Macmillan (2009). p. 193–206.

[6] Grewal I Lewis J Flynn T Brown J Bond J Coast J. Developing attributes for a generic quality of life measure for older people: Preferences or capabilities? Soc Sci Med. (2006) 62:1891–901. 10.1016/j.socscimed.2005.08.023 16168542

[7] Sutton EJ Coast J. Development of a supportive care measure for economic evaluation of end-of-life care using qualitative methods. Palliat Med. (2014) 28:151–7. 10.1177/0269216313489368 23698452

[8] Brazier J Connell J Papaioannou D Mukuria C Mulhern B Peasgood T. A systematic review, psychometric analysis and qualitative assessment of generic preference-based measures of health in mental health populations and the estimation of mapping functions from widely used specific measures. Health Technol Assess. (2014) 18:vii–viii, xiii–xxv:1–188. 10.3310/hta18340 24857402 PMC4781324

[9] Mulhern B Mukuria C Barkham M Knapp M Byford S Soeteman Dr. Using generic preference-based measures in mental health: psychometric validity of the EQ-5D and SF-6D. Br J Psychiatry. (2014) 205:236–43. 10.1192/bjp.bp.112.122283 24855127

[10] Davis S Wailoo A. A review of the psychometric performance of the EQ-5D in people with urinary incontinence. Health Qual Life Outcomes. (2013) 11:20. 10.1186/1477-7525-11-20 23418844 PMC3622573

[11] Yang Y Brazier J Longworth L. EQ-5D in skin conditions: an assessment of validity and responsiveness. Eur J Health Econ. (2015) 16:927–39. 10.1007/s10198-014-0638-9 25358263 PMC4646948

[12] Haywood KL Garratt AM Fitzpatrick R. Quality of life in older people: a structured review of generic self-assessed health instruments. Qual Life Res. (2005) 14:1651–68. 10.1007/s11136-005-1743-0 16119178

[13] Kuspinar A Mayo NE. A review of the psychometric properties of generic utility measures in multiple sclerosis. Pharmacoeconomics. (2014) 32:759–73. 10.1007/s40273-014-0167-5 24846760

[14] Tordrup D Mossman J Kanavos P. Responsiveness of the EQ-5D to clinical change: is the patient experience adequately represented? Int J Technol Assess Health Care. (2014) 30:10–9. 10.1017/S0266462313000640 24499622

[15] Hounsome N Orrell M Edwards RT. EQ-5D as a quality of life measure in people with dementia and their carers: evidence and key issues. Value Health. (2011) 14:390–9. 10.1016/j.jval.2010.08.002 21402307

[16] Li L Nguyen KH Comans T Scuffham P. Utility-based instruments for people with dementia: a systematic review and meta-regression analysis. Value Health. (2018) 21:471–81. 10.1016/j.jval.2017.09.005 29680105

[17] Finch AP Brazier JE Mukuria C. What is the evidence for the performance of generic preference-based measures? A systematic overview of reviews. Eur J Health Econ. (2018) 19:557–70. 10.1007/s10198-017-0902-x 28560520 PMC5913394

[18] Payakachat N Ali MM Tilford JM. Can the EQ-5D detect meaningful change? A systematic review. Pharmacoeconomics. (2015) 33:1137–54. 10.1007/s40273-015-0295-6 26040242 PMC4609224

[19] Moher D Shamseer L Clarke M Ghersi D Liberati A Petticrew M. Preferred reporting items for systematic review and meta-analysis protocols (PRISMA-P) 2015 statement. Syst Rev. (2015) 4:1. 10.1186/2046-4053-4-1 25554246 PMC4320440

[20] Terwee CB Jansma EP Riphagen II de Vet HCW. Development of a methodological PubMed search filter for finding studies on measurement properties of measurement instruments. Qual Life Res. (2009) 18:1115–23. 10.1007/s11136-009-9528-5 19711195 PMC2744791

[21] Mokkink LB Terwee CB Patrick DL Alonso J Stratford PW Knol DL. The COSMIN study reached international consensus on taxonomy, terminology, and definitions of measurement properties for health-related patient-reported outcomes. J Clin Epidemiol. (2010) 63:737–45. 10.1016/j.jclinepi.2010.02.006 20494804

[22] Mokkink LB de Vet HCW Prinsen CAC Patrick DL Alonso J Bouter LM. COSMIN risk of bias checklist for systematic reviews of patient-reported outcome measures. Qual Life Res. (2018) 27:1171–9. 10.1007/s11136-017-1765-4 29260445 PMC5891552

[23] Terwee CB Bot SD de Boer MR van der Windt DA Knol DL Dekker J. Quality criteria were proposed for measurement properties of health status questionnaires. J Clin Epidemiol. (2007) 60:34–42. 10.1016/j.jclinepi.2006.03.012 17161752

[24] Prinsen CAC Vohra S Rose MR Boers M Tugwell P Clarke M. How to select outcome measurement instruments for outcomes included in a “Core Outcome Set” – a practical guideline. Trials. (2016) 17:449. 10.1186/s13063-016-1555-2 27618914 PMC5020549

[25] Prinsen CAC Mokkink LB Bouter LM Alonso J Patrick DL de Vet HCW. COSMIN guideline for systematic reviews of patient-reported outcome measures. Qual Life Res. (2018) 27:1147–1157. 10.1007/s11136-018-1798-3 29435801 PMC5891568

